# Evidence for strong environmental control on bacterial microbiomes of Antarctic springtails

**DOI:** 10.1038/s41598-021-82379-x

**Published:** 2021-02-03

**Authors:** Chiara Leo, Francesco Nardi, Claudio Cucini, Francesco Frati, Peter Convey, James T. Weedon, Dick Roelofs, Antonio Carapelli

**Affiliations:** 1 Life Science Department, University of Siena, Via Aldo Moro 2, 53100 Siena, Italy; 2 British Antarctic Survey, Natural Environment Research Council, High Cross, Madingley Road, Cambridge, CB3 0ET UK; 3 Department of Ecological Sciences, Vrije Universiteit Amsterdam, De Boelelaan 1081, 1081 HV Amsterdam, The Netherlands; 4 Keygene N.V., Agro Business Park 90, 6708 PW Wageningen, The Netherlands; 5 Department of Life Sciences, Imperial College London, London, UK

**Keywords:** Evolution, Zoology, Ecology, Biodiversity, Biogeography, Microbiology, Microbial communities

## Abstract

Collembola are a key component of the soil biota globally, playing an important role in community and ecosystem dynamics. Equally significant are their associated microbiomes, that can contribute to key metabolic functions. In the present study, we investigated the bacterial community composition of four Antarctic springtail species to assess if and how the extreme Antarctic environment has shaped the collembolans’ microbiomes. Springtails were collected from two biogeographical regions, the maritime and the continental Antarctic. From each region, two endemic species, belonging to the genera *Cryptopygus* (Isotomidae, Entomobryomorpha) and *Friesea* (Neanuridae, Poduromorpha), were included. This experimental design allowed us to quantify the relative importance of ecological factors (different regions of occurrence) and/or phylogenetic divergence in the host (different Orders) in shaping the Collembola microbiome. The diversity and richness of springtail microbiomes was lower in the Antarctic taxa compared to published information from species from temperate regions. The microbiome composition was predominantly species-specific, with a limited core microbiome shared across the four species examined. While both geographic origin and host species influenced the associated microbiomes, the former was the prevalent driver, with closer similarity between springtails from the same bioregion than between those belonging to the same genus.

## Introduction

The Collembola (springtails) is one of the richest taxa of basal Hexapoda and, due to their important role in soil ecosystem functioning, one of the most studied components of the soil fauna. Since springtails can feed on fungal hyphae, bacteria, algae, mosses, spores and decaying organic matter, their effects on soil dynamics can be direct (e.g., by producing/feeding on organic matter) or indirect (e.g., by influencing microbial activity and community composition by grazing)^1–6^. Despite the importance of springtails for soil dynamics, few studies to date have investigated their associated microbiomes, thus limiting our knowledge of the various metabolic, physiological and immunological functions to which the microbiota may contribute. In this respect, the study of the microbiomes associated with Antarctic springtails, adapted to extreme and almost pristine environments, may provide an important advance in understanding of bacterial contribution to springtail biology and, in turn, to Antarctic terrestrial ecosystem functioning.

Antarctic terrestrial ecosystems experience some of the harshest conditions on our planet, with habitats (permanently or seasonally snow- and ice-free areas) limited to less than 0.3% of the continent’s area^7–10^. Three main biogeographic regions are generally recognised in Antarctica: the sub-, maritime and continental Antarctic^7^. The latter two experience the harshest climatic conditions, with chronically low to very low temperatures, often low annual precipitation and limited availability of liquid water^7–9^.

Examination of Antarctic springtail microbiomes may shed new light on the evolutionary adaptation to the abiotic stresses that typify Antarctica’s extreme and isolated ecosystems^9,11^, as well as allowing the identification of currently unknown biotic associations. Existing studies of springtail microbiomes have suggested that they are characterised by the dominance of Acidobacteria, Actinobacteria, Bacteroidetes, Firmicutes and Proteobacteria^12–15^. It has also been suggested that some of these taxa may play a role in antibiotic production, the provision of metabolic pathways for nitrogen and terpene metabolism, and the production of chitinases necessary for the digestion of food components^12–14^. Nevertheless, the application of Next Generation Sequencing (NGS) techniques to study springtail-associated microbiomes has to date mostly focused on model species from temperate environments, such as *Folsomia candida*^12,13^, *Orchesella cincta*^14^, *Folsomia quadrioculata*^15^ and other Entomobryomorpha^16^. However, Collembola display substantial evolutionary diversity and, during their long evolutionary history, have adapted to occupy almost every terrestrial ecosystem on Earth, including Antarctica’s frigid deserts^5^. There is therefore a need to expand knowledge of their microbiomes to include additional taxa adapted to different ecological conditions.

In this study, we characterized the microbiomes associated with different Antarctic springtail species using the V3 region of the bacterial 16S ribosomal RNA (rRNA) encoding gene as a molecular marker. Four springtail species, belonging to two different genera from different Orders, were selected from two different Antarctic Conservation Biogeographic Regions (ACBRs^17,18^). Two species, *Cryptopygus antarcticus antarcticus* (Willem, 1901) and *Friesea antarctica* (Willem, 1901) (until recently known as *Friesea grisea*^19^) were collected in ACBR3 North-West Antarctic Peninsula, specifically from the South Shetland Islands and Adelaide Island (both in the maritime Antarctic). *Cryptopygus terranovus* (Wise, 1967) and *F. propria* (Greenslade and Fanciulli, 2020) (also previously known as *F. griesea*^20^), were sampled from ACBR8 North Victoria Land (continental Antarctica).

All four species are endemic to different regions within Antarctica and, given their poor dispersal capabilities and the occurrence of insurmountable geographical barriers, have been isolated in these regions for many millions of years during their evolution^20–23^. Therefore, studying their microbiomes may allow robust evaluation of the extent to which Antarctic environments have influenced the springtail-associated bacterial communities, compared with springtails from lower latitudes. The simple two-factor sampling design used in this study may also allow differentiation of two potential major evolutionary drivers responsible for shaping Collembola microbiomes in Antarctica. Specifically, that of the phylogenetic similarity of hosts (through comparing two pairs of congeneric species), *versus* that of two distinct biogeographic regions (maritime *vs* continental Antarctic).

## Results

### Data processing

Sequencing produced 259,086–327,432 reads per replicate (see “Materials and methods” section for naming conventions). After demultiplexing and trimming, the number of reads imported into QIIME2 ranged between 129,543 and 163,716. Quality pre-processing and chimera filtering, along with sequence pair joining, further reduced the number of paired reads per replicate to 54,045–89,636. Removal of mitochondrial and chloroplast sequences in QIIME2, as well as the manual removal of co-amplified springtail 18S sequences, generated a final dataset of 1318–17,740 sequences per replicate. It is notable that a high number of sequences resulting from co-amplification of springtail 18S were obtained, accounting for 73–96% of sequences per replicate. This is partly explained by the high nucleotide similarity observed at primer binding sites with springtail 18S sites (in the springtail families Isotomidae and Neanuridae in general), which may have been exacerbated during amplification. Finally, OTU clustering led to the identification of a total of 1026 bacterial OTUs, ranging between 46 and 116 OTUs per replicate.

### Diversity of Antarctic springtail microbiome

**Figure 1:**
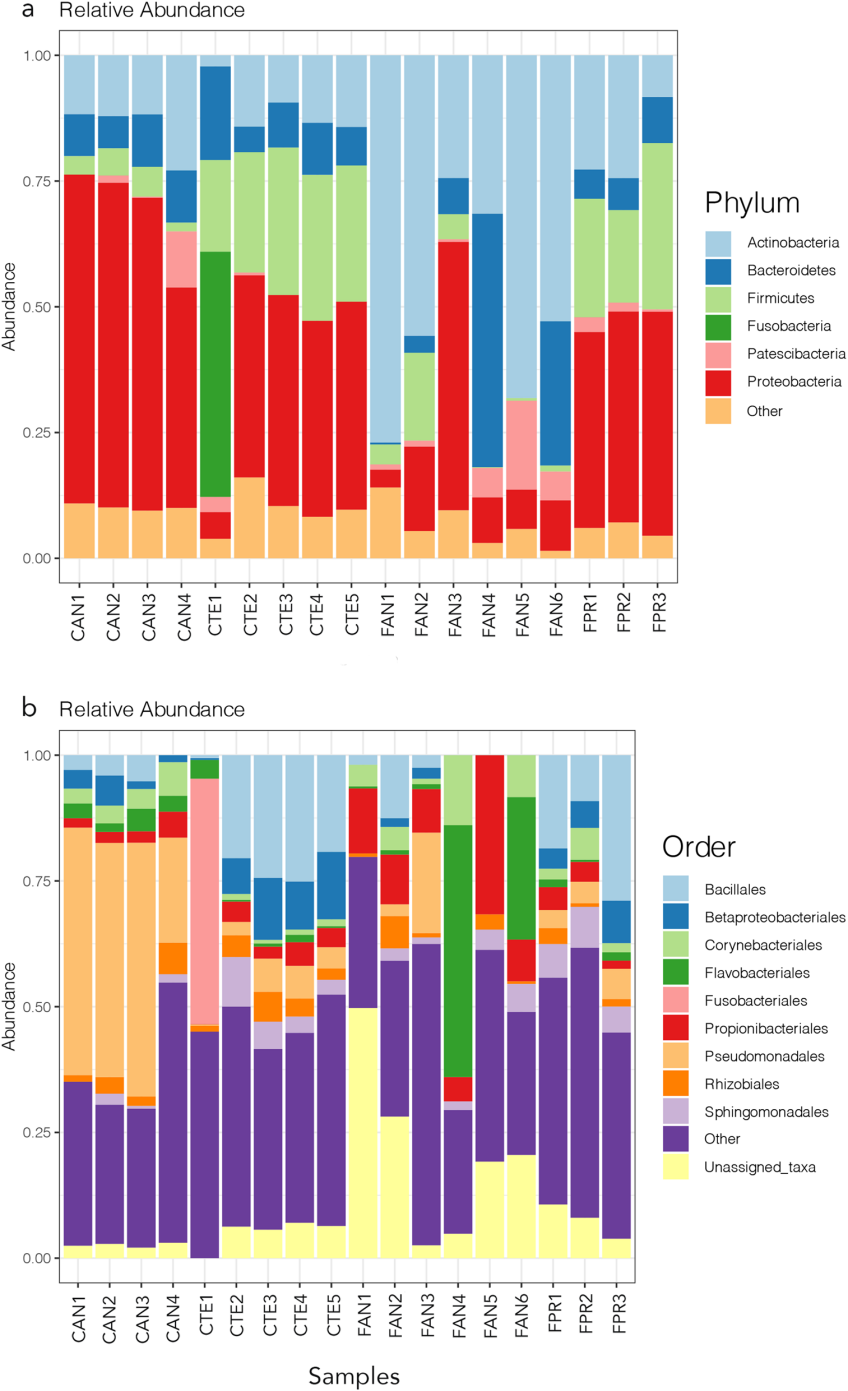
Relative abundance plots of the most frequent bacterial OTUs at the phylum (**a**) and order (**b**) levels. Samples are grouped according to host species and sampling location, see Table 1 for explanation of codes. The figure was generated using the R package ggplot2 v3.2.1 (https://ggplot2.tidyverse.org).

Among the 1026 identified bacterial OTUs, the dominant higher taxa were Proteobacteria (average relative frequency 33%), Actinobacteria (27%), Firmicutes (14%) and Bacteroidetes (11%) (Fig. 1a).

**Figure 2:**
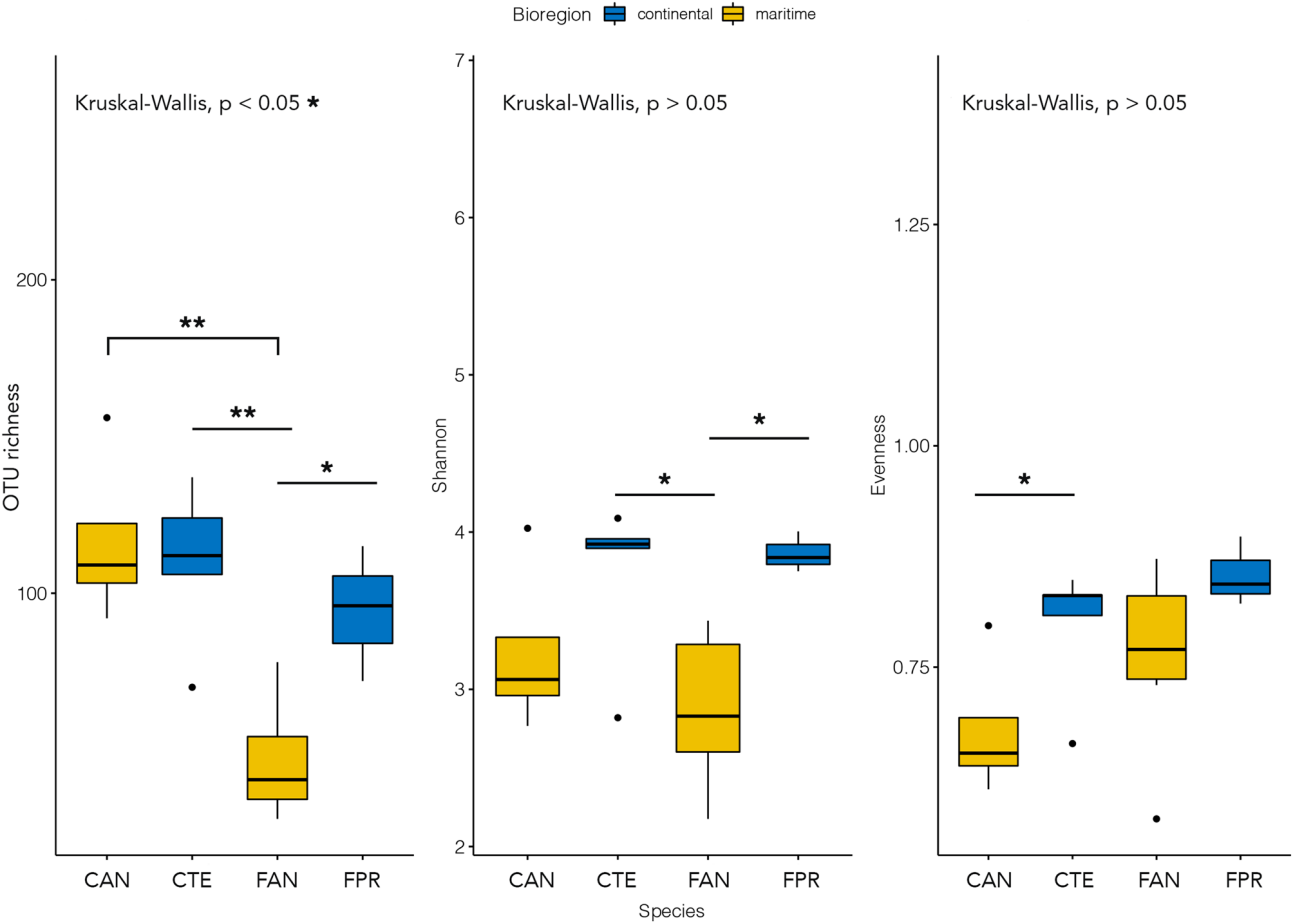
Alpha diversity: observed OTU richness, Shannon and Evenness indexes. The P value of the Kruskal–Wallis test is shown at the top of each plot; lines and asterisks highlight significant differences observed after pairwise Mann–Whitney–Wilcoxon tests. *P < 0.05; **P < 0.01. Blue and yellow colours indicate species collected in the continental and maritime Antarctic regions, respectively. The figure was generated using the R package ggplot2 v3.2.1 (https://ggplot2.tidyverse.org).

The alpha diversity described by the Shannon and the Evenness indexes was characterised by moderate levels of bacterial diversity and the absence of dominant OTUs across all the samples analysed (Fig. 2). The overall observed number of OTUs per sample was 94 ± 33 (mean ± standard deviation), with the lowest value detected in *F. antarctica* (56 ± 22; Fig. 2). The OTU richness in the *F. antarctica* microbiome was significantly different from all others in pairwise comparisons performed using the Wilcoxon rank sum test (P < 0.05; Fig. 2; Supplementary Table S1). The Shannon metric showed a moderate intrinsic diversity in each microbial community examined, with an average of 3.44 ± 0.55 (Fig. 2). The intrinsic diversity described by the Shannon index was significantly different between the microbiomes of springtails distributed across the two Antarctic bioregions, with Shannon indices being on average 3.78 ± 0.40 and 3.16 ± 0.49 for continental and maritime Antarctic, respectively (P < 0.05, Supplementary Table S1). The Evenness index was moderately high among all samples, suggesting a balanced distribution of taxa (on average 0.78 ± 0.09). A significant difference was observed in the Wilcoxon test between the two *Cryptopygus* species sampled in the two Antarctic bioregions, whose Evenness indices were 0.68 ± 0.08 and 0.80 ± 0.08 for *C. a. antarcticus* and *C. terranovus*, respectively (P < 0.05, Fig. 2; Supplementary Table S1).

**Figure 3:**
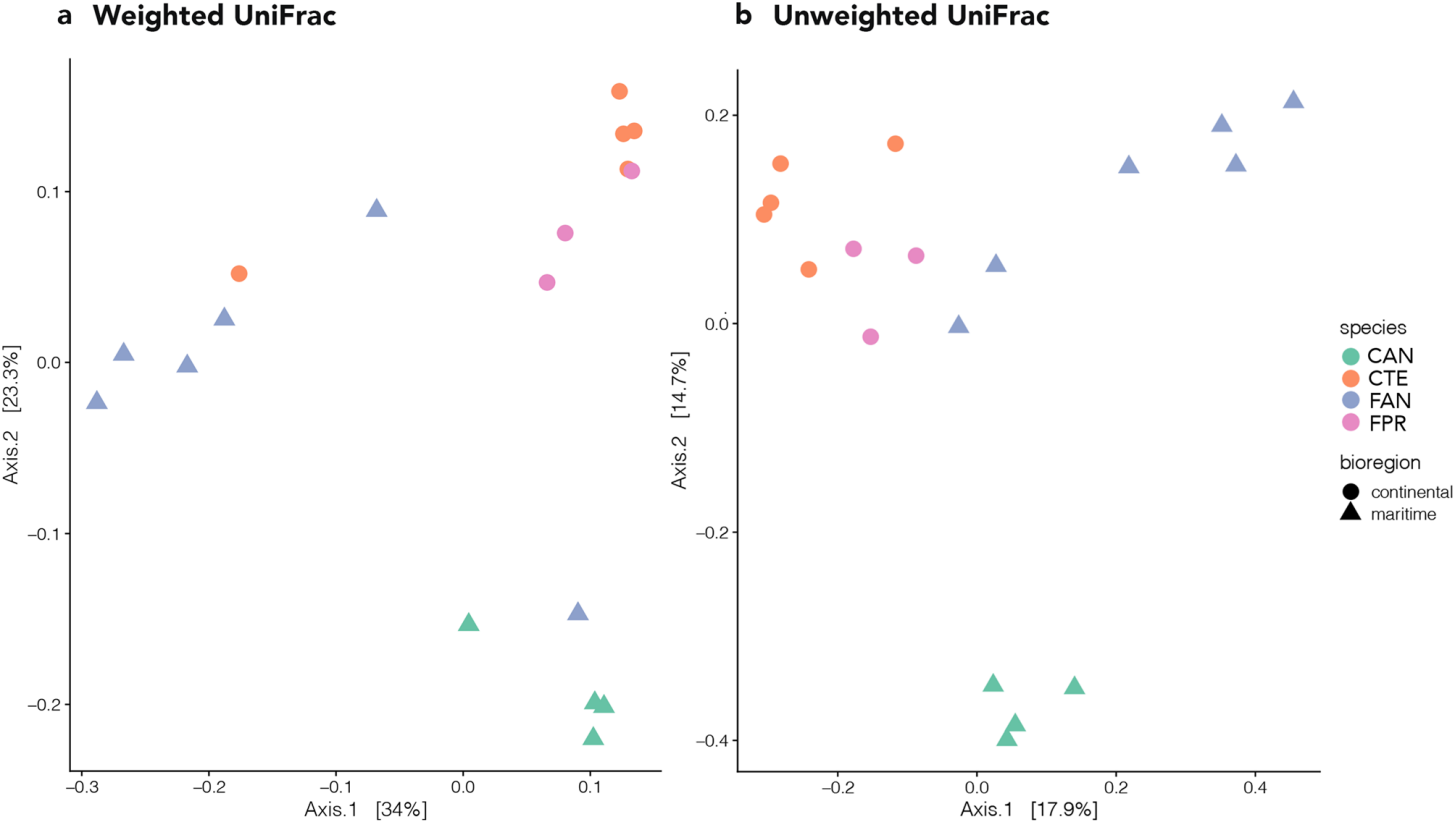
Principal coordinate analysis (PCoA) showing: (**a**) the weighted UniFrac, and (**b**) the unweighted UniFrac distances between the four samples analysed. The figure was generated using the R package ggplot2 v3.2.1 (https://ggplot2.tidyverse.org).

Beta diversity was explored using two phylogeny-based methods, weighted and unweighted UniFrac distances, with the former focusing on phylogenetic distance and frequency of OTUs (i.e., quantitative) and the latter on phylogenetic distance only (i.e., qualitative). In the Principal Coordinates Analysis (PCoA) based on weighted UniFrac distances, the first axis (PCoA1) explained 34% of variation and the second (PCoA2) explained 23.3% (Fig. 3a). The first axis showed a slight difference in composition between *F. antarctica* and all other samples. The second axis separated *C. a. antarcticus* from all the other samples and, in general, it tended to cluster the microbiomes according to their hosts’ geographic distribution (maritime *vs* continental Antarctic; Fig. 3a). PERMANOVA analysis on weighted UniFrac distances indicated that differences were significant at all levels, i.e., between species, between genera (both P < 0.001) and between bioregions (P < 0.05; Supplementary Table S2).

The PCoA performed using unweighted UniFrac distances showed clear distinction in OTU composition between the four springtail microbiomes (Fig. 3b). The first axis (PCoA1) explained 17.9% of variation and tended to separate samples according to bioregion (i.e., maritime *vs* continental Antarctic; Fig. 3b). The second axis (PCoA2) explained 14.7% of variation and overall separated the *C. a. antarcticus* replicates from the remaining samples (Fig. 3b). Additional axes explained a sizeable portion of the observed variance (axis 3: 9%, axis 4: 7.9%), but groupings did not allow for a clear biological interpretation (data not shown). The PERMANOVA analysis again indicated that differences were statistically significant between species and genera (both P < 0.001), as well as bioregions (P < 0.05; Supplementary Table S2).

**Figure 4:**
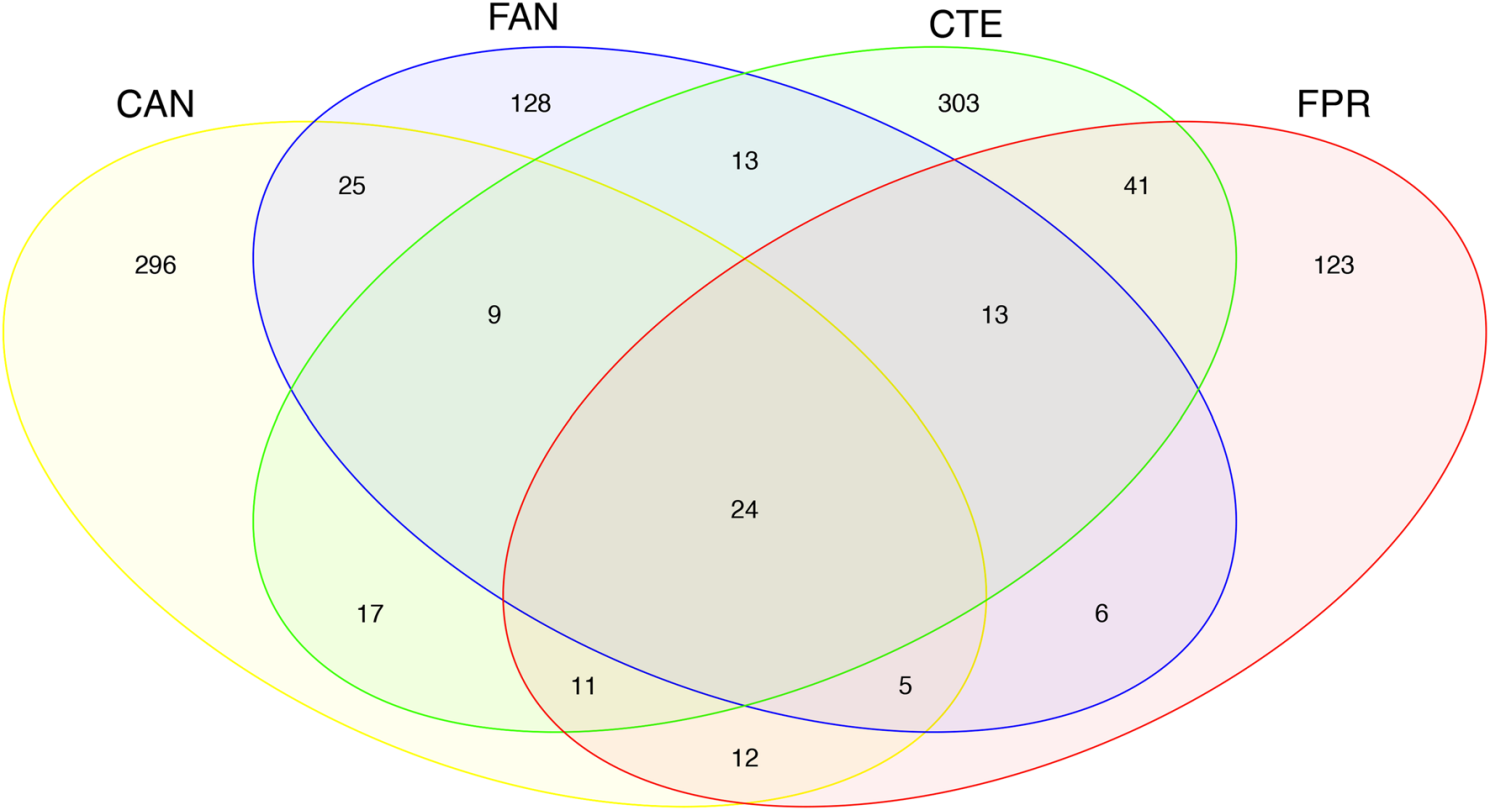
Venn diagram showing the shared and unique OTUs of the microbiomes associated with the four Antarctic springtail species. Sample labels are expanded in Table 1. The figure was generated using the R package gplots v3.0.1.1 (https://CRAN.R-project.org/package=gplots).

The core microbial community associated with the four springtail species was investigated through a Venn diagram. Among the 1026 OTUs detected, only 24 were present in all samples (Fig. 4). These latter were OTUs belonging to the phyla Actinobacteria, Deinococcus-Thermus, Firmicutes, Lentisphaerae and Proteobacteria, as well as, when properly classified, belonging to the most abundant orders shown in Fig. 1b. The number of shared OTUs increased when considering the core microbiome of samples grouped by biogeographic region of origin or by genus. In particular, *C. a. antarcticus* and *F. antarctica* from the maritime Antarctic had 63 OTUs in common, while *C. terranovus* and *F. propria* from Victoria Land shared 89 OTUs. In comparison, the two *Cryptopygus* species shared 61 OTUs and the two *Friesea* species shared 48 OTUs.

## Discussion

Previous studies of the microbiota associated with Antarctic fauna, such as the oribatid mite *Alaskozetes antarcticus*^24^ and the tardigrade *Acutuncus antarcticus*^25^, showed higher diversity than those reported in mite and tardigrade species from lower latitudes. This was not observed in Antarctic springtail microbiomes. On the one hand, the most abundant bacterial groups, herein identified at the phylum level, were consistent with the microbial community composition reported in previous studies of Collembola microbiomes (Fig. 1a^12,14^). The Antarctic springtail microbiomes were dominated by Proteobacteria, Bacteroidetes, Firmicutes and Actinobacteria (Fig. 1a), taxa that have been previously suggested to be mainly involved in decomposition processes, as well as possibly providing a defence system through antimicrobial production^12–14^. Although at high taxonomic levels the dominant microbiota appears to be similar between temperate and Antarctic Collembola, more pronounced differences exist at lower taxonomic levels. However, these are difficult to interpret at present in biological terms, as both the precise taxonomy and the metabolic functions of individual species are mostly unknown.

Interestingly, the overall OTU richness observed in this study, seems to be considerably lower than that described for the temperate springtails *Folsomia candida* and *Orchesella cincta* (ca. 200–300 OTUs per sample^12,14^), although some uncertainty may result from unequal sequencing depth. These data would suggest that, as reported for macro-organisms, the richness of bacterial species associated with Antarctic springtails decreases at higher latitudes. A similar pattern was observed in a comparison of the bacterial community composition of Antarctic and Australian soils^26^, although not in a comparison of the diversity of Arctic and other Northern Hemisphere soils^27^. Antarctic soils are characterized by lower levels of organic carbon, as a result of lower temperatures a more limited vegetation, than those of the Arctic^23–25,28^. Multiple factors therefore appear to underlie reduced diversity of microorganisms in Antarctic soils. We suggest that the same environmental factors may have acted over evolutionary time to limit the diversity of Antarctic springtail microbiomes compared with those of Collembola from lower latitudes.

Along with ecosystem characteristics, the influence of the host taxon may be equally important in defining host-microbiota relationships. This has been reported in studies of tardigrades from low and high latitudes, that showed microbiome composition to be highly species-specific^25^, and may also be the case in the Antarctic springtails studied here, in which the numbers of OTUs unique to each sample outnumbered those shared (Fig. 4). This suggestion is further corroborated by the PERMANOVA analyses, where significant divergence between microbiomes was detected across all comparisons (Supplementary Table S2).

The data obtained in the present study also support a major influence of environmental factors in shaping microbiome composition. While this result appears plausible, as it is based on the comparison of two pairs of species, some caution is needed in the interpretation of data from the *C. a. antarcticus/C. terranovus* species pair, as the former has been reared under laboratory conditions for 18 months before the study^25,29^. This can account for reduction and/or modification of the microbial diversity compared to natural populations (e.g.,^29^), as shown in a comparison of whole-body microbiomes between natural and laboratory-reared populations of the Antarctic tardigrade *Acutuncus antarcticus*^25^.

Analyses of beta diversity identified a higher similarity between the microbiomes associated with different springtail genera originating in the same Antarctic bioregion than with species belonging to the same genus but originating from different bioregions (Fig. 4). The number of shared OTUs between *C. a. antarcticus* and *F. antarctica* (63) (maritime Antarctic), and between *C. terranovus* and *F. propria* (89) (continental Antarctic) were higher than the numbers shared between species belonging to the same genus (i.e., 61 and 48 shared OTUs between *Cryptopygus* and *Friesea* species, respectively; Fig. 4). Although it has been observed that phylogenetically related springtails play similar roles in foodwebs and occupy similar ecosystem niches^30^, it seems that the composition of their microbiomes, that should be—in some respects—linked to their role in soil dynamics, does not follow the evolutionary niche conservation principle. Moreover, Collembola have evolved various feeding strategies, leading to distinct and specialized morphology of mouthparts, probably to avoid niche overlap when different springtails species are present in the same ecosystem^5^. In spite of this, the results obtained suggest a higher similarity in the microbiota composition between springtail species belonging to different genera, families and orders (that may be characterised by different feeding behaviours). Therefore, it appears that the phylogenetic relatedness of hosts seems not to be the primary driver affecting their associated bacterial community composition, and this latter should be rather connected with their high degree of endemism and the long-term survival of hosts in a given biogeographical region of Antarctica. This evidence contrasts with the ubiquity hypothesis of microbial distribution and, as other studies have suggested^31,32^, refutes the idea that for microbes “everything is everywhere”^33^.

It is also possible that the extreme conditions experienced in Antarctica mean that environmental control is so pronounced that it has become the primary factor shaping not only the evolution and adaptation of both Antarctic terrestrial prokaryotes and invertebrate fauna, but also their physiological and ecological interactions. Furthermore, the long separation time between biota of the continental Antarctic and maritime Antarctic, from few millions to many tens of millions of years (e.g.,^34^), may have further fostered the development of highly specific microbial interactions. This may be a direct consequence of time, with divergence in the associated microbial community paralleling divergence in the hosts. Such differential selection, arising from diverse environmental conditions, may have led to the development of novel and varied ecological interactions (i.e., being in contact with different microbial assemblages in the soil of two distinct regions may have opened to the possibility of establishing novel and different host-microbiota relationships).

## Conclusions

The present study presents the first characterization of the microbiomes associated with two pairs of congeneric Antarctic springtail species originating from two distinct biogeographical regions (the maritime and continental Antarctic). These regions experience very different environmental conditions that strongly impact on the composition and functioning of their terrestrial ecosystems. Moreover, their microbiota and invertebrate fauna have long evolutionary histories in isolation^20,34,35^. The reduced diversity and richness of Antarctic springtail microbiomes, as well as the limited similarities observed between the four microbiomes analysed here, could be the result of strong environmental control, as well as of millions of years of evolutionary separation. Future application of metagenomic functional analyses will allow a better understanding of the evolution of host-microbiome relationships and their contribution to host adaptation to extreme environmental conditions, as well as of the metabolic and physiological properties arising from these interactions.

## Materials and methods

### Sample collection

The samples analysed in the present study were collected from the two main Antarctic biogeographical regions, the maritime and continental Antarctic (Table 1). In the former, samples were obtained from Livingston Island (South Shetland Islands) and Lagoon Island, Ryder Bay (off the south-east coast of Adelaide Island). In the latter, samples were collected from Campo Icaro and Kay Island, Northern Victoria Land. Specimens of *F. antarctica* were sampled during the austral summer of 2002–2003 in collaboration between the British Antarctic Survey (BAS) and the Italian National Antarctic Program (PNRA). Specimens of *C. terranovus* and *F. propria* from Victoria Land were collected during PNRA-supported expeditions in the austral summers of 2017–2018 and 2018–2019, respectively. Samples of *C. a. antarcticus,* originating from the Antarctic Peninsula, were collected in January 2018 and reared in a substrate of Antarctic mosses in a climate chamber at + 2 °C from July 2018, in the Department of Systems Ecology of the Vrije Universiteit Amsterdam.

**Table 1:** List of the species included in the present study.

Replicate ID	Species	Sample coordinates	Collection sites	Bioregion
CTE1	*Cryptopygus terranovus*	Campo Icaro 74°42′45"S; 164° 06′21"E	Ice-free rocky ground devoid of vegetation	Continental Antarctic; North Victoria Land (ACBR 8)
CTE2				
CTE3				
CTE4				
CTE5				
CAN1	*Cryptopygus antarcticus antarcticus*	South Shetland Islands 62°0′0″S; 58°0′0″W	Reared in climatic chamber (+ 2 °C) on Antarctic mosses since July 2018	Maritime Antarctic; North-West Antarctic Peninsula (ACBR 3)
CAN2				
CAN3				
CAN4				
FPR1	*Friesea propria*	Kay Island 74°04′14"S; 165°18′60"E	Rocky ground on slope with sparse vegetation	Continental Antarctic; North Victoria Land (ACBR 8)
FPR2				
FPR3				
FAN1	*Friesea antarctica*	Devils Point Livingston Island South Shetland Islands 62°40′15″S; 61°10′57″W	Rocky ground close to seashore with sparse vegetation	Maritime Antarctic; North-West Antarctic Peninsula (ACBR 3)
FAN2				
FAN3				
FAN4		Lagoon Island Adelaide Island 67°35′33″S; 68°14′06″W	Rocky ground close to seashore	
FAN5				
FAN6				

The number of replicates per species, as well as the collection site, the geographical coordinates and the bioregion are given.

Throughout the text we use the term “replicate” to refer to individual DNA isolates, while the terms “sample” or “species” are used to designate groups of replicates from the same springtail species collected in the same sampling location (two neighbouring sampling locations in the case of *F. antarctica*). In the analyses described below two types of comparisons are made, (1) partitioning samples across “region” (continental Antarctic *vs.* maritime Antarctic) and (2) across “genera” (i.e., *C. terranovus* and *C. a. antarcticus vs. F. propria* and *F. antarctica*).

### DNA extraction and sequencing

Between 15 and 20 animals were pooled, rinsed in 70% ethanol and their DNA extracted using the QIAamp UCP DNA Micro kit (Qiagen, Hilden, Germany) following the manufacturer’s instructions. A minimum of three replicates for each species and location were included in the study (see Table 1 for details). A first PCR was performed, using primers 341F (5′-CCTACGGGNGGCWGCAG-3′) and 518R (5′-ATTACCGCGGCTGCTGG-3′). The amplification was carried out in a total reaction volume of 25 μl, consisting of 0.5 μl of deoxynucleotides (dNTPs), 1.25 μl of each primer (10 μM), 5 μl of 5× Phusion High-Fidelity Buffer, 0.5 μl of Phusion Hot-Start High-Fidelity Taq polymerase (Thermo Fischer Scientific Inc., Waltham, MA, USA), 13.5 μl of ddH_2_O and 3 μl of template. The following cycling conditions were applied: denaturation at 98 °C for 10 s, annealing at 50 °C for 30 s and elongation at 72 °C for 30 s, repeated for 25 cycles; an initial denaturation was performed at 98 °C for 2 min, as well as a final elongation step at 72 °C for 10 min. As longer non-specific products may potentially have been co-amplified, the expected ~ 170 bp 16S target was purified from the gel slice using the Nucleospin Gel kit and PCR Clean-Up (Macherey-Nagel, Düren, Germany) following the manufacturer’s protocol. Purified PCR products were re-amplified with primers linked to Illumina adapters with the same reaction mixture following a 12 cycle program: denaturation at 98 °C for 10 s, annealing and extension at 72 °C for 1 min, as well as an initial denaturation at 98 °C for 2 min and a final elongation at 72 °C for 8 min. PCR products were visualized on a 1.5% agarose gel and purified using Beckman Coulter Agencourt AMPure XP protocols, with a ratio DNA:beads of 1:1. Purified products were quantified using the Qubit dsDNA HS Assay Kit (Thermo Fischer Scientific Inc., Waltham, MA, USA). Samples were then indexed using Herculase II Fusion DNA Polymerase Nextera XT Index Kit V2 and sequenced on a MiSeq Illumina platform at the Macrogen Inc. facility (Seoul, Republic of Korea).

All amplicon data are available from the NCBI Sequence Read Archive (SRA) database under the project number PRJNA681366 (https://www.ncbi.nlm.nih.gov/sra/PRJNA681366).

### Sequence processing

Demultiplexed raw sequences were trimmed to remove 16S primers using Trimmomatic v0.39^36^. Raw sequences were imported into the program Quantitative Insights Into Microbial Ecology v2 (QIIME2^37^). Sequences were filtered and merged using the DADA2 plugin, truncating the forward and the reverse reads after 230 and 188 bp, respectively, based on the read quality^38^. Sequences were clustered using a 97% identity threshold with VSEARCH^39^, including chimera removal. Taxonomic identification of the OTUs was performed with the feature-classifier plugin trained on the SILVA v132 database^40^. OTUs classified as mitochondrial or chloroplast were removed, as well as sequences corresponding to the springtail ribosomal 18S gene. A phylogenetic analysis was carried out using the maximum likelihood optimization criterion, as implemented in the phylogeny plugin, using the fasttree method^41^. Alpha and beta diversity were computed in R v3.6.1 (2019-07-05), using the R packages microbiome^42^, microbiomeSeq^43^, phyloseq v1.28^44^, vegan v2.5-6^45^ and limma v3.40.6^46^, after data normalization to a sampling depth of 1318 (the minimum number of reads observed in the smallest library; see Table 1). To investigate alpha diversity, the observed number of OTUs per normalized sample, as well as the Shannon and Evenness indices, were calculated. Differences between groups were tested using both the Kruskal–Wallis and Wilcoxon rank sum tests (equivalent to the Mann–Whitney test; the significance level was defined as α  = 0.05). P values were adjusted for multiple comparisons in both tests using the Holm–Bonferroni method. Comparison between samples (beta diversity) were conducted based on phylogenetic UniFrac weighted and unweighted distances. A PERMANOVA analysis, performing 999 permutations and setting the level of significance as α = 0.05, was applied to the weighted and unweighted distance matrices to test for differences in beta diversity. Comparisons were carried out at the levels of species, genera and biogeographic region (maritime vs. continental Antarctic; Table 1). Sharing of OTUs was investigated through a Venn diagram using the R package VennDiagram^47^ on the original and not normalized OTU table. Finally, the data were visualized using the R packages ggplot2 v3.2.1^48^ and gplots 3.0.1.1^49^. These two latter tools were applied to generate the figures shown in the present paper.

## Supplementary Information

Supplementary Tables.
