# Supplementary material for: Evidence for strong environmental control on bacterial microbiomes of Antarctic springtails
Source: Sci Rep. 2021 Feb 3;11:2973. doi: 10.1038/s41598-021-82379-x (PMC7858589; doi:10.1038/s41598-021-82379-x)
Supplement: Supplementary file 1 — Supplementary Tables. [file 41598_2021_82379_MOESM1_ESM.pdf]

Evidence for strong environmental control on bacterial microbiomes of Antarctic springtails

Chiara Leo[1,§,*], Francesco Nardi[1], Claudio Cucini[1], Francesco Frati[1], Peter Convey[2], James T. Weedon[3], Dick Roelofs[4], Antonio Carapelli[1]

[1] University of Siena, Life Science Department, Via Aldo Moro 2, Siena, 53100, Italy

[§] Current address: Department of Life Sciences, Imperial College London, London, UK:

[2] British Antarctic Survey, Natural Environment Research Council, High Cross, Madingley Road, Cambridge, CB3 0ET, United Kingdom

[3] Vrije Universiteit Amsterdam, Department of Ecological Sciences, De Boelelaan 1081, Amsterdam, 1081 HV, The Netherlands

[4] Keygene N.V., Agro Business Park 90, Wageningen, 6708 PW, The Netherlands

* leo6@student.unisi.it

Index	Group 1	Group 2	p value	p adjusted	p format	p signif
**Between species**						
OTU richness	CAN	CTE	1,0000	1,0000	1,0000	ns
	CAN	FAN	0,0095	0,0520	0,0095	**
	CAN	FPR	0,6286	1,0000	0,6286	ns
	CTE	FAN	0,0087	0,0520	0,0087	**
	CTE	FPR	0,5714	1,0000	0,5714	ns
	FAN	FPR	0,0476	0,1900	0,0476	*
**Between genera**						
OTU richness	*Cryptopygus*	*Friesea*	0,0056	0,0056	0,0056	**
**Between bioregions**						
OTU richness	maritime	continental	0,0831	0,0830	0,0830	ns
	**Kruskal-Wallis test**					
OTU richness			0,0145	0,0150	0,0150	*

Index	Group 1	Group 2	p value	p adjusted	p format	p signif
Between species						
Shannon	CAN	CTE	0,4127	1,0000	0,4130	ns
	CAN	FAN	0,3524	1,0000	0,3520	ns
	CAN	FPR	0,4000	1,0000	0,4000	ns
	CTE	FAN	0,0303	0,1500	0,0300	*
	CTE	FPR	0,7857	1,0000	0,7860	ns
	FAN	FPR	0,0238	0,1400	0,0240	*
**Between genera**						
Shannon	*Cryptopygus*	*Friesea*	0,1903	0,1900	0,1900	ns
**Between bioregions**						
Shannon	maritime	continental	0,0117	0,0120	0,0120	*
	**Kruskal-Wallis test**					
Shannon			0,0595	0,0590	0,0590	ns

Index	Group 1	Group 2	p value	p adjusted	p format	p signif	method
Between species							
Evenness	CAN	CTE	0,0317	0,1900	0,0320	*	Wilcoxon
	CAN	FAN	0,3524	1,0000	0,3520	ns	
	CAN	FPR	0,0571	0,2900	0,0570	ns	
	CTE	FAN	0,6623	1,0000	0,6620	ns	
	CTE	FPR	0,3929	1,0000	0,3930	ns	
	FAN	FPR	0,2619	1,0000	0,2620	ns	
**Between genera**							
Evenness	*Cryptopygus*	*Friesea*	0,3401	0,3400	0,3400	ns	Wilcoxon
**Between bioregions**							
Evenness	maritime	continental	0,0434	0,0430	0,0430	*	Wilcoxon
	**Kruskal-Wallis test**						
Evenness			0,0886	0,0890	0,0890	ns	

Supplementary Table S1: Results of the Kruskal-Wallis and Wilcoxon rank sum tests. The significance level was defined as α= 0.05. The P values were adjusted for multiple comparisons using the Holm-Bonferroni method.

Permanova Weighted UniFrac				Permanova Unweighted UniFrac			
**Between Species**				**Between Species**			
Df	3	14	17	Df	3	14	17
SumsOfSqs	0,561	0,641	1,203	SumsOfSqs	1,871	3,505	5,376
MeanSqs	0,187	0,046	-	MeanSqs	0,624	0,250	-
F Model	4,086	-	-	F Model	2,491	-	-
R2	0,467	0,533	1,000	R2	0,348	0,652	1,000
Pr F	0,001***	-	-	Pr F	0,001***	-	-
**Between genera**				**Between genera**			
Df	1	16	17	Df	1	16	17
SumsOfSqs	0,270	0,933	1,203	SumsOfSqs	0,778	4,598	5,376
MeanSqs	0,270	0,058	-	MeanSqs	0,778	0,287	-
F Model	4,623	-	-	F Model	2,707	-	-
R2	0,224	0,776	1,000	R2	0,145	0,855	1,000
Pr F	0,001***	-	-	Pr F	0,001***	-	-
**Between bioregions**				**Between bioregions**			
Df	1	16	17	Df	1	16	17
SumsOfSqs	0,164	1,039	1,203	SumsOfSqs	0,627	4,749	5,376
MeanSqs	0,164	0,065	NA	MeanSqs	0,627	0,297	NA
F Model	2,518	NA	NA	F Model	2,111	NA	NA
R2	0,136	0,864	1,000	R2	0,117	0,883	1,000
Pr F	0,02*	NA	NA	Pr F	0,003***	NA	NA

Supplementary Table S2: Detailed results of the PERMANOVA analyses performed using the weighted (left panel) and the unweighted (right panel) UniFrac beta diversity distances. The significance level was defined as α= 0.05.
